# Susceptibility of *Lutzomyia longipalpis* (Lutz & Neiva, 1912) to Fludora FusionPM, a combination of clothianidin and deltamethrin: field and laboratory bioassays

**DOI:** 10.1186/s13071-025-07206-y

**Published:** 2025-12-24

**Authors:** Greicielly Barbosa Santos Silva, Josiane Valadão Lopes, Grasielle Caldas D’ Ávila Pessoa, Leticia Cavalari Pinheiro, João Paulo dos Santos, Fabiana Oliveira Lara-Silva, Nadja Biondine Marriel, Liléia Gonçalves Diotaiuti, Consuelo Latorre Fortes-Dias, Edelberto Santos Dias

**Affiliations:** 1 Laboratório de Leishmanioses, Instituto René Rachou (FIOCRUZ Minas), Av. Augusto de Lima 1715, Belo Horizonte, MG CEP 30190-009 Brazil; 2 Departamento de Parasitologia, Instituto de Ciências Biológicas, Universidade Federal de Minas Gerais (UFMG), Av. Antônio Carlos, 6627, Belo Horizonte, MG CEP 31270-901 Brazil; 3 Núcleo de Estudos em Saúde Pública e Envelhecimento (NESPE), Instituto René Rachou (FIOCRUZ Minas), Av. Augusto de Lima 1715, Belo Horizonte, MG CEP 30190-009 Brazil; 4 Unidade de Controle de Zoonoses, Secretaria Municipal de Saúde, Rua Bom Jesus s/n, Jaboticatubas, MG CEP 35830-000 Brazil; 5 Laboratório de Referência em Triatomíneos e Epidemiologia da Doença de Chagas, Instituto René Rachou (FIOCRUZ Minas), Av. Augusto de Lima 1715, Belo Horizonte, MG CEP 30190-009 Brazil; 6 Diretoria de Pesquisa e Desenvolvimento, Fundação Ezequiel Dias (FUNED), Rua Conde Pereira Carneiro 80, Belo Horizonte, MG CEP 30550-010 Brazil

**Keywords:** Phlebotomine sand fly, Chemical control, Leishmaniasis, Insecticide, Pyrethroid, Neonicotinoid

## Abstract

**Background:**

Insecticides remain a cornerstone in the control of vector-borne diseases. In Brazil, Alfatek 200SC—a pyrethroid (alphacypermethrin)—is recommended for controlling phlebotomine sand flies, the vectors of leishmaniases. For mosquitoes such as *Aedes aegypti*, a combination of deltamethrin and clothianidin (Fludora FusionPM) is endorsed. This study evaluated the efficacy of Fludora FusionPM against phlebotomine sand flies.

**Methods:**

Laboratory bioassays were conducted using Fludora FusionPM-impregnated filter papers stored either at room temperature (25 ± 2 °C) or under cold conditions (3 ± 1 °C). In field trials, painted (PS) or unpainted (US) masonry-plastered walls in selected households were treated with the insecticide. Alfatek 200SC was used as a reference, following the same procedures. *Lutzomyia longipalpis* females (*n* = 25 per replicate, in triplicate) were exposed for 60 min using the cone test. Mortality was assessed 1 h and 24 h postexposure and quarterly over 1 year. Structured questionnaires were administered to the community endemic agent (CEA) responsible for spraying and to household residents to document perceived adverse effects.

**Results:**

In the laboratory, the residual activity expressed by average mortality rates over 1 year of paper impregnation was of 97.6% for Fludora FusionPM) and 91.7% for Alfatek 200SC). In the field, 1-year average mortality rates were 97.3% for Fludora FusionPM and 94.6% for Alfatek 200SC for PS. On US walls, Fludora^®^ FusionPM maintained high mortality rate (97.8%) whereas Alfatek 200SC parameter decreased to 80.7%. Adverse effects were informed by three of five residents for Alfatek 200SC and by one of four residents for Fludora FusionPM. The CEA reported side reactions after Alfatek 200SC spraying.

**Conclusions:**

Fludora FusionPM was highly toxic to *Lu. longipalpis* and outperformed Alfatek 200SC under field conditions, particularly on unpainted masonry-plastered walls. Combining insecticides with complementary modes of action may enhance rotational strategies, reduce costs and resistance risk, and optimize control of multiple vector-borne diseases simultaneously.

**Graphical abstract:**

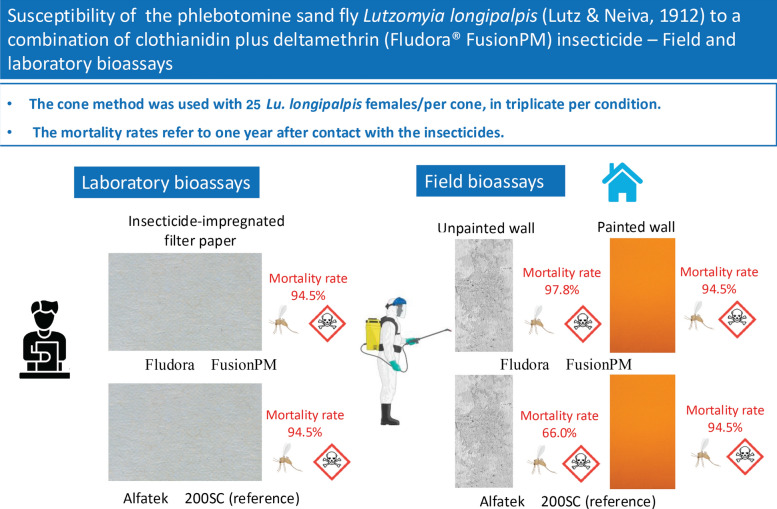

**Supplementary Information:**

The online version contains supplementary material available at 10.1186/s13071-025-07206-y.

## Background

Insecticides are widely and effectively used to control vector-borne diseases such as leishmaniasis. Indoor residual spraying is among the World Health Organization’s top recommendations for controlling visceral leishmaniasis (VL), as it reduces phlebotomine sand fly populations and consequently decreases transmission to humans [1]. In this context, accurate data on the susceptibility of sand flies to available insecticides are essential for designing effective control strategies.

In Brazil, the primary vector of VL is *Lutzomyia longipalpis* (Diptera: Psychodidae: Phlebotominae), which is widely distributed across the country [2]. The pronounced endophilic behavior of *Lu. longipalpis* supports the potential for intrahousehold transmission of VL, justifying the application of insecticides in residential settings [3, 4]. Currently, pyrethroids are the main class of insecticides used against phlebotomine sand flies, with alpha-cypermethrin being the most widely applied in control programs. Pyrethroids disrupt the insect nervous system by affecting voltage-gated sodium channels, resulting in prolonged depolarization, paralysis, and ultimately death [5, 6].

In 2018, a formulation combining deltamethrin and clothianidin—marketed as Fludora FusionPM (Bayer CropScience)—was prequalified by the WHO for indoor spraying against mosquito populations [7]. Deltamethrin is a pyrethroid ester, while clothianidin belongs to the neonicotinoid class of nicotine-derived insecticides. Neonicotinoids act as agonists at insect acetylcholine receptors (nAChRs), causing hyperexcitation of the nervous system. Clothianidin is effective against a broad spectrum of insect species, exhibits low mammalian toxicity, and does not show cross-resistance, making it an ideal candidate for integrated vector management [8]. In 2020, this dual-action insecticide was officially adopted in Brazil for the chemical control of *Aedes aegypti*, the vector of dengue [9]. With the ongoing introduction of new active ingredients in vector control programs, it is crucial to assess the susceptibility and potential resistance of phlebotomine sand flies to the dual insecticide.

It is important to note that the long-term success of vector control programs relies not only on the effectiveness of the insecticides employed but also on the implementation of proactive insecticide resistance management (IRM) strategies. Continuous use of a single active ingredient or chemical class imposes strong selection pressure on vector populations, favoring the emergence and spread of resistant phenotypes. To mitigate this risk, it is strongly recommended to rotate active ingredients and/or to use insecticides with different modes of action, thereby reducing the likelihood of selecting resistant populations [10, 11]. In this context, the availability of effective alternatives to alpha-cypermethrin 20% SC is essential to ensure the sustainability of control measures in areas where resistance may develop or be detected.

To the best of our knowledge, there are no published data on the susceptibility of phlebotomine sand flies to Fludora FusionPM. To fill this gap, we conducted laboratory and field bioassays using a model population of *Lu. longipalpis*, comparing the results with those obtained using alpha-cypermethrin (the reference insecticide and positive control). The residual efficacy of both products was monitored over 1 year. The findings may enlighten new strategies for the prevention and control of visceral leishmaniasis.

## Methods

### Insecticides

Fludora FusionPM (Bayer CropScience), a wettable powder combining 6.25% deltamethrin and 50.0% clothianidin (plus 43.75% inert ingredients), was applied at 100 g/10 L of water (0.4 g/m^2^). Alfatek 200SC, containing 20% alpha-cypermethrin (plus 80% of inert ingredients), at 50 mL/10 L (40 mg a.i./m^2^), was used as reference (positive control). Type I water served as negative control.

### Sand fly colony

F1 to F5 females of *Lutzomyia longipalpis*, 2- to 4-days old were obtained from a colony of the Reference Center for Vector Competence of Sand Flies (RCVCS) at the René Rachou Institute (Lapinha Cave origin, SISBIO license no. 18728-1). This colony is highly susceptible to alpha-cypermethrin [12]. The sand flies were maintained at 26.6 ± 1 °C and 80 ± 10% humidity, with ad libitum 50% sugar solution before and after the bioassays.

### Laboratory bioassays

**Fig. 1:**
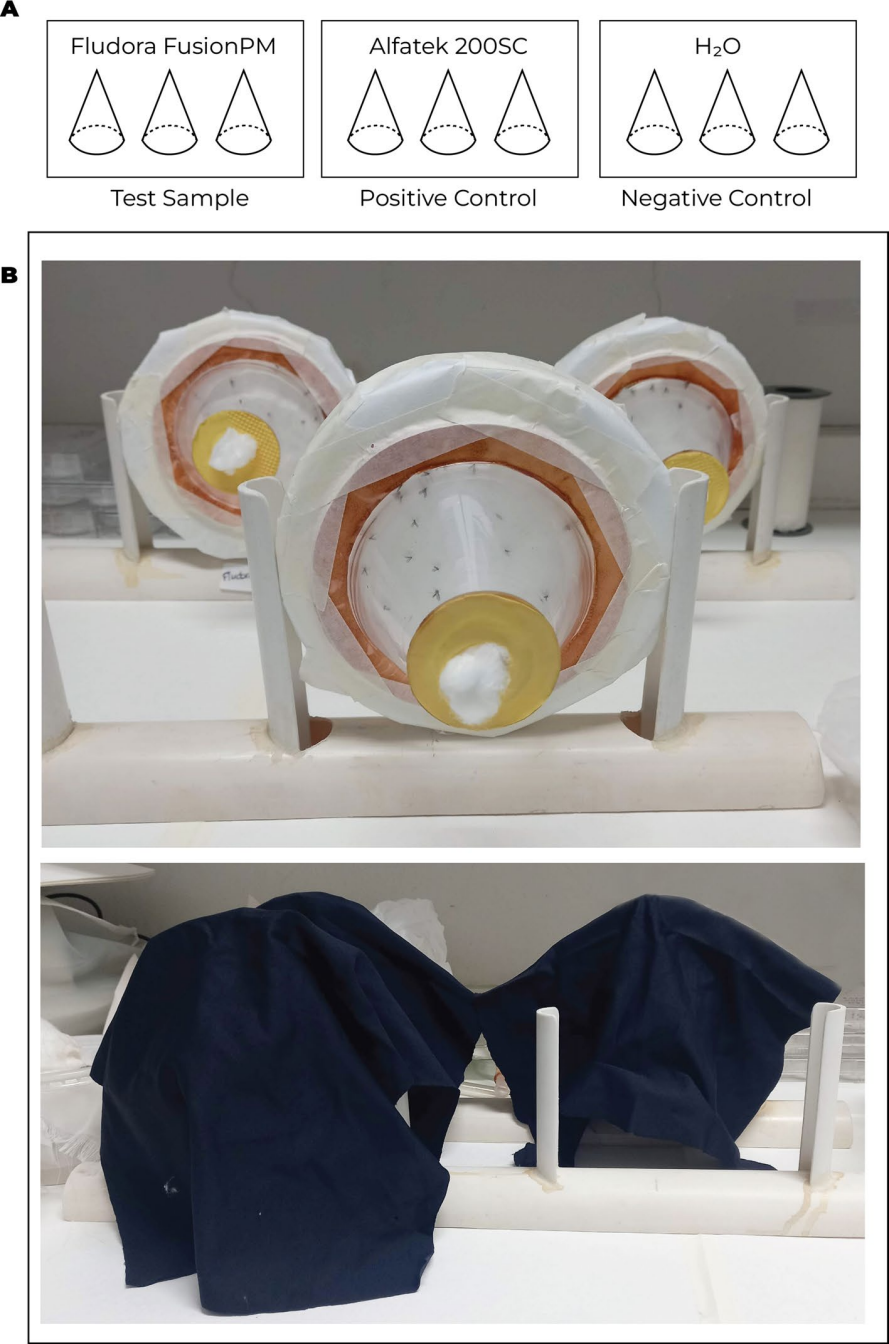
Laboratory bioassay to test the susceptibility of *Lu. longipalpis* to insecticides. **A** The experimental design included a test sample of Fludora FusionPM, a positive control of Alfatek 200SC, and a negative control (type I water). **B** Modified acrylic cones were covered with black cloth after 25 females of *Lu. longipalpis* were placed inside. Exposure time lasted 60 min. Photo credits: GBS Silva

Laboratory bioassays began in September 2022 and continued through October 2023. Filter papers (18 × 18 cm) were impregnated with insecticide using a micropipette (12.96 mg of Fludora FusionPM in 1.30 mL water, or 3.24 µL of Alfatek 200SC in 1.30 mL water). Papers were wrapped in aluminum foil and stored at room temperature (25 ± 2 °C) or refrigerated (3 ± 1 °C). Custom acrylic WHO cones with rubber seals were used to accommodate the small sand fly size (Fig. 1). Each replicate included 25 females per cone, with three replicates per treatment. Exposure lasted 60 min.

### Field bioassays

The assays were conducted from February 2023 to February 2024 in six household units (HUs) in Jaboticatubas (19°30′49″S; 43°44′42″W), an area endemic for VL in the state of Minas Gerais, Brazil. The municipality is located at a suitable distance from the laboratory (66 km) to ensure the health and integrity of the phlebotomine sand flies during transportation to the field and back to the laboratory. Households had no prior insecticide application and the residents consented to the single indoor spraying at the beginning of the study and the indoor access every 3 months until 1 year.

**Fig. 2:**
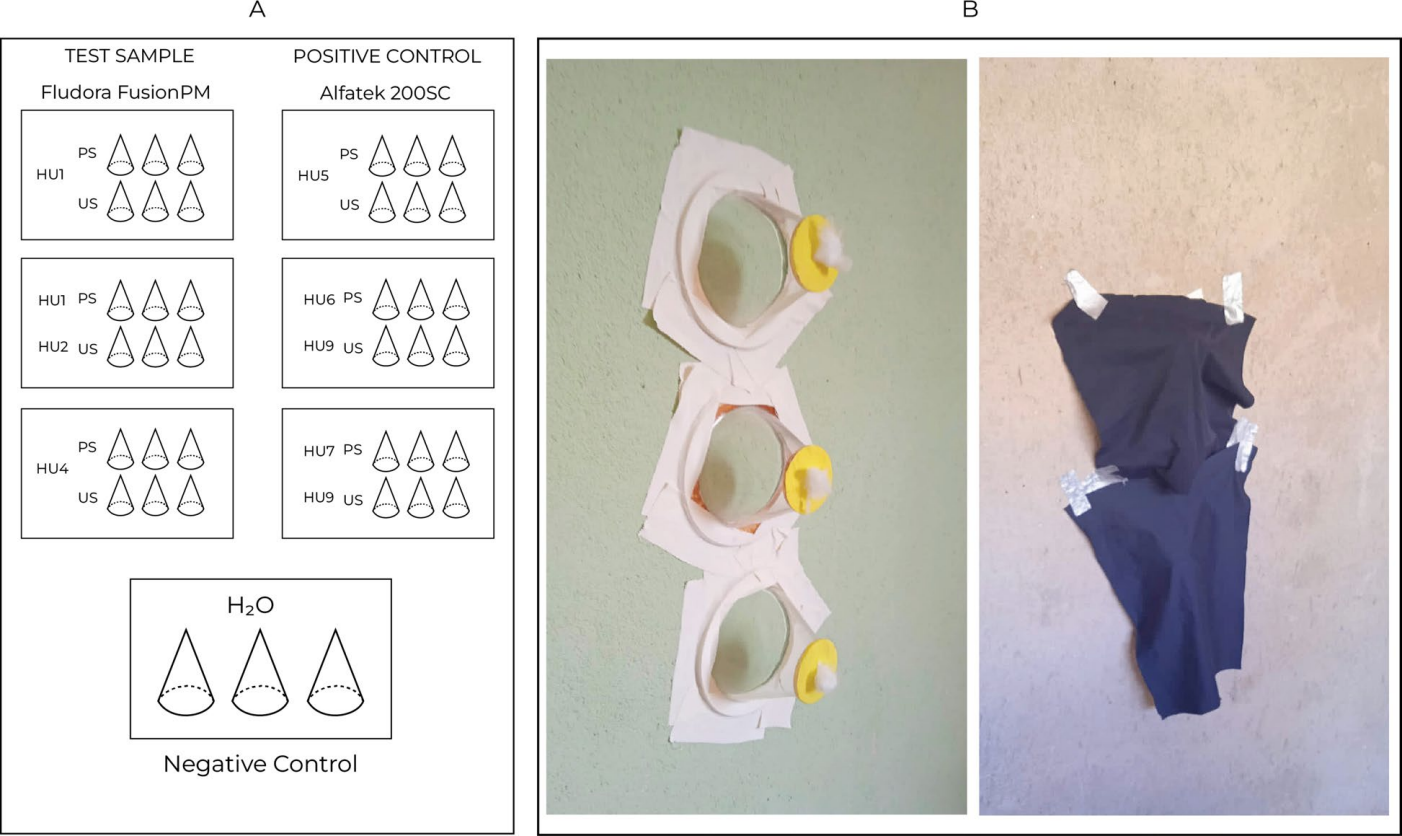
Field bioassay design to assess *Lu. longipalpis* susceptibility to Fludora FusionPM. **A** The experimental design included a test sample of Fludora FusionPM, a positive control of Alfatek 200SC, and a negative control (untreated wall surface). *Hun* household unit followed by identification number (*n*), *PS* painted masonry-plastered surface, *US* unpainted masonry-plastered surface. **B** Wall surfaces were treated with Fludora FusionPM (test sample), Alfatek 200SC (positive control), or not-treated (negative control). Cones were stapled to the surfaces and each cone was populated with 25 females of *Lu. longipalpis* and enveloped with black cloth, followed by a contact period of 60 min. Photo credits: GBS Silva

Three PS (painted) and three US (unpainted) masonry-plastered walls were insecticide-treated (Fig. 2). Negative control was untreated filter paper in smooth surface.

Indoor spraying was performed by a trained Community Endemic Agent (CEA) from the municipal Zoonosis Control Center (CCZ), according to the protocol of the Program of Surveillance and Control of Visceral Leishmaniasis (PSCVL) of the Ministry of Health of Brazil [13]. The sprayings used calibrated equipment (10 L capacity, Tee Jet 8002E nozzle, 25–55 lbs compression) and were operated by the same CEA to mitigate potential biases in the outcomes. Vertical stripes (75 cm wide, 5 cm overlap) were sprayed alternately top-to-bottom, 45 cm from the wall, at 2 s per meter. The spraying occurred in February of 2023 with the bioassays starting 24 h postspraying. Cones were attached vertically, 25 females introduced, and the whole apparatus was covered with black cloth. Exposure lasted 60 min.

### Mortality assessment

Mortality was calculated by dead specimens or as the percentage (mortality rate) of dead flies per cone. Numbers from each set of three cones were pooled, whenever necessary. Flies unable to stand or showing uncoordinated movements were counted as dead; those with coordinated flight were alive. After initial assessment, flies were transferred to clean Styrofoam boxes with untreated plaster pots and maintained under colony conditions. Mortality was reassessed 24 h postexposure to account for the breakdown effect. *Lutzomyia longipalpis* mortality was evaluated quarterly for 1 year in both laboratory and field trials. The parameter was used to evaluate the degree of sand fly susceptibility as well as the residual effect of the insecticide.

The results were expressed according to the specified time point (1 h, 24 h; 0, 3, 6, 9, or 12 months), overtime (0, 3, 6, 9, and 12 months), after 1 year of impregnation, or as the average of all the reading times (1-year average).

### Statistical analysis

Analyses were performed in R [14] at a 5% significance level. Nonparametric tests were applied (Kruskal–Wallis followed by Dunn’s post-test Bonferroni correction for comparisons among more than two independent samples, and the Mann–Whitney *U* test for comparisons between two independent samples—using overtime readings. Regression curves were fitted and statistically compared using GraphPad Prism v.10.6.1 at a 5% significance level.

### Ethical considerations

The study was approved by the René Rachou Institute Ethics Committee, the FIOCRUZ Animal Use Committee (License LW-15/16), and the Animal Experimentation Ethics Commission. Procedures followed WHO guidelines [15]. Informed consent was obtained from household owners, and spraying was announced 96 h and 24 h in advance. Residents received guidance on preparation, protection, and posterior cleaning. Semistructured questionnaires were answered by one resident per HU (*n* = 9) and by the ACE, 24 h after spraying completion.

## Results

### Laboratory bioassays

**Fig. 3:**
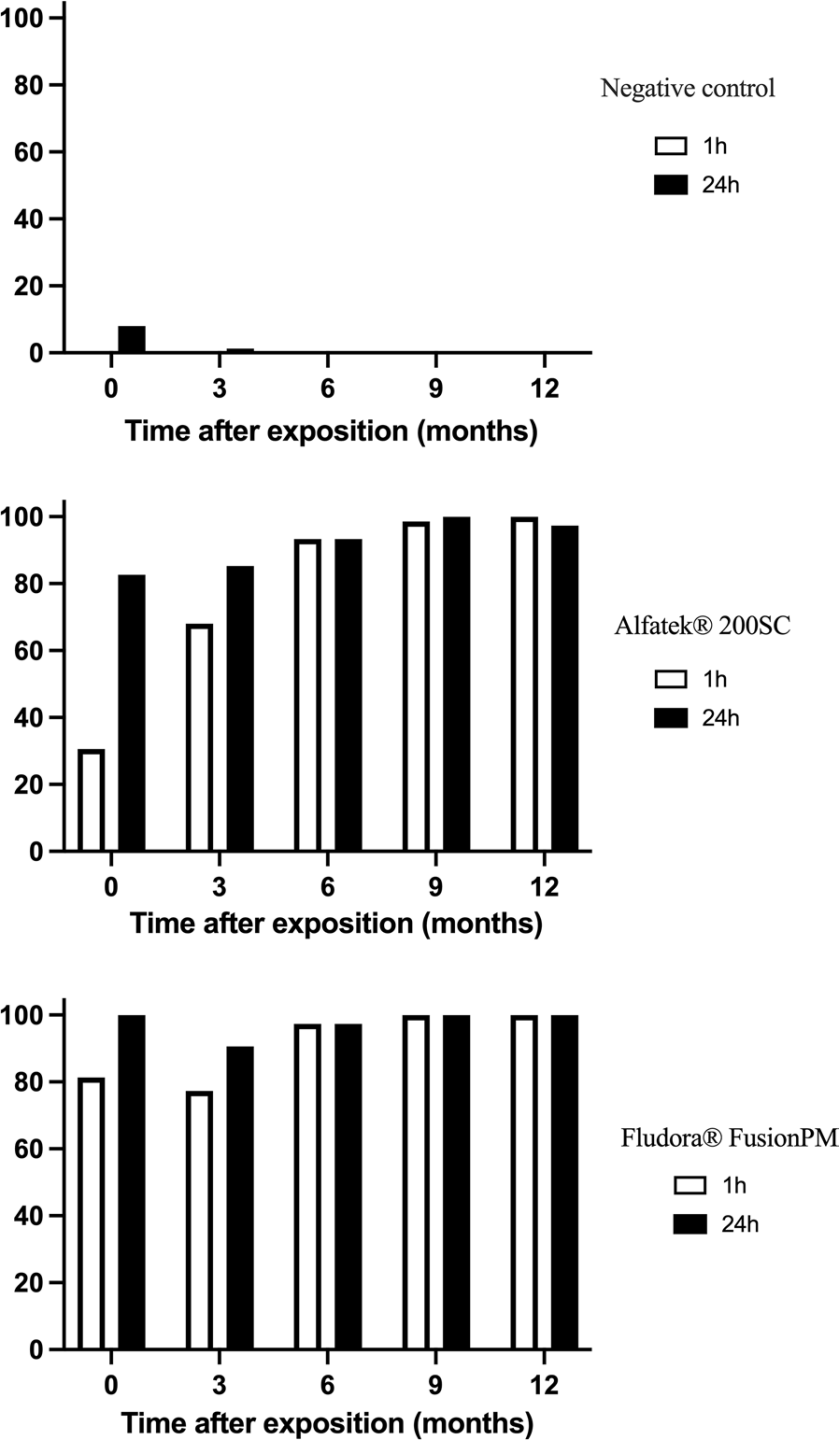
Susceptibility of *Lu. longipalpis* to Fludora FusionPM at specified time points in laboratory bioassays. Mortality rates were assessed after 1 h (white bars) and 24 h (black bars) of exposure via cone assay.A total of 25 *Lu. longipalpis* females were transferred per cone, in a total of 75 specimens per test sample (triplicate). Impregnated papers were stored at room temperature, protected from light. Data collected from September 2022 to October 2023

At time zero and 1-hour reading, Fludora FusionPM was more effective than Alfatek 200SC, with mortality rates of 81.3% (61/75) and 30.6% (23/75), respectively (Fig. 3). At the 24 h-reading, the mortality rate to Alfatek 200SC increased to 82.7% (62/75), whereas Fludora FusionPM reached 100.0% (75/75), confirming the importance of delayed mortality assessment (24 h) in such bioassays.

Mortality rates at the 24-h readings of *Lu. longipalpis* exposed to Fludora FusionPM or Alfatek 200SC were compared with the negative control using the Kruskal–Wallis test. A statistically significant difference was detected among at least two of the three groups (*H* = 33.275, df = 2, *P* < 0.0001). Subsequent pairwise comparisons using Dunn’s post-test with Bonferroni correction showed that mortality in the negative control was significantly lower than in both insecticide groups (Fludora FusionPM vs. negative control (*Z* = −5.588, *P* < 0.0001); Alfatek 200SC versus negative control (*Z* = 4.034, *P* = 0.0002). In contrast, the comparison between Fludora FusionPM and Alfatek 200SC (*Z* = −1.554, *P* = 0.361) indicated no significant difference in mortality, demonstrating that both insecticides were similarly effective against *Lu. longipalpis*. Only 24 h readings were considered in subsequent analyses.

The same figure (Fig. 3) illustrates the effective residual effect of the insecticides. At 1 year after the exposition, the mortality rates remained 100% (75/75) for Fludora FusionPM and 97.3% (73/75) for Alfatek 200SC. The 1-year average mortality rates stayed in 97.6% (366/375) for Fludora FusionPM and 91.7% (344/375) for Alfatek 200SC.

**Fig. 4:**
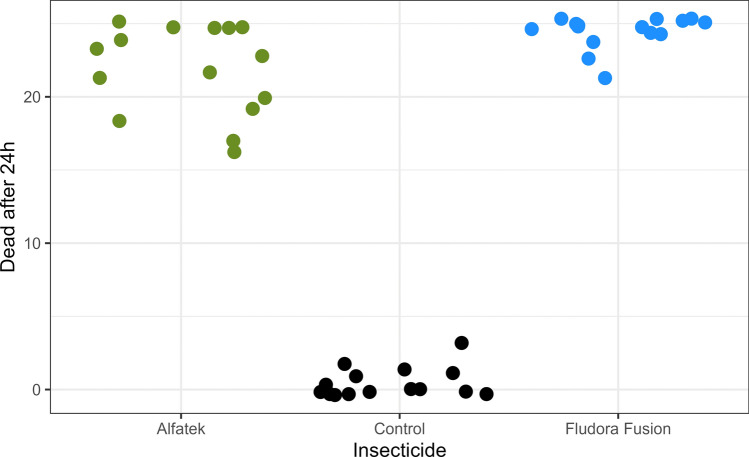
Mortality rates of *Lu. longipalpis* females over time, after exposition to Fludora FusionPM via cone assay in the laboratory. Each dot represents one cone containing 25 specimens of *Lu. longipalpis* females, totaling 15 cones (triplicates across five timepoints: 0, 3, 6, 9, and 12 months). Alfatek 200SC served as the reference (positive control). X-axis represent the insecticide or negative control. Legend: Black dots- negative control; blue dots- Fludora FusionPM; green dots- Alfatek 200SC. Impregnated papers were stored at room temperature. Period: September 2022 to October 2023

Data dispersion for the insecticide effect is represented in Fig. 4. Fludora FusionPM results were more consistent as observed by higher concentration and lesser dispersion of data points in the upper, right quadrant of the figure.

**Fig. 5:**
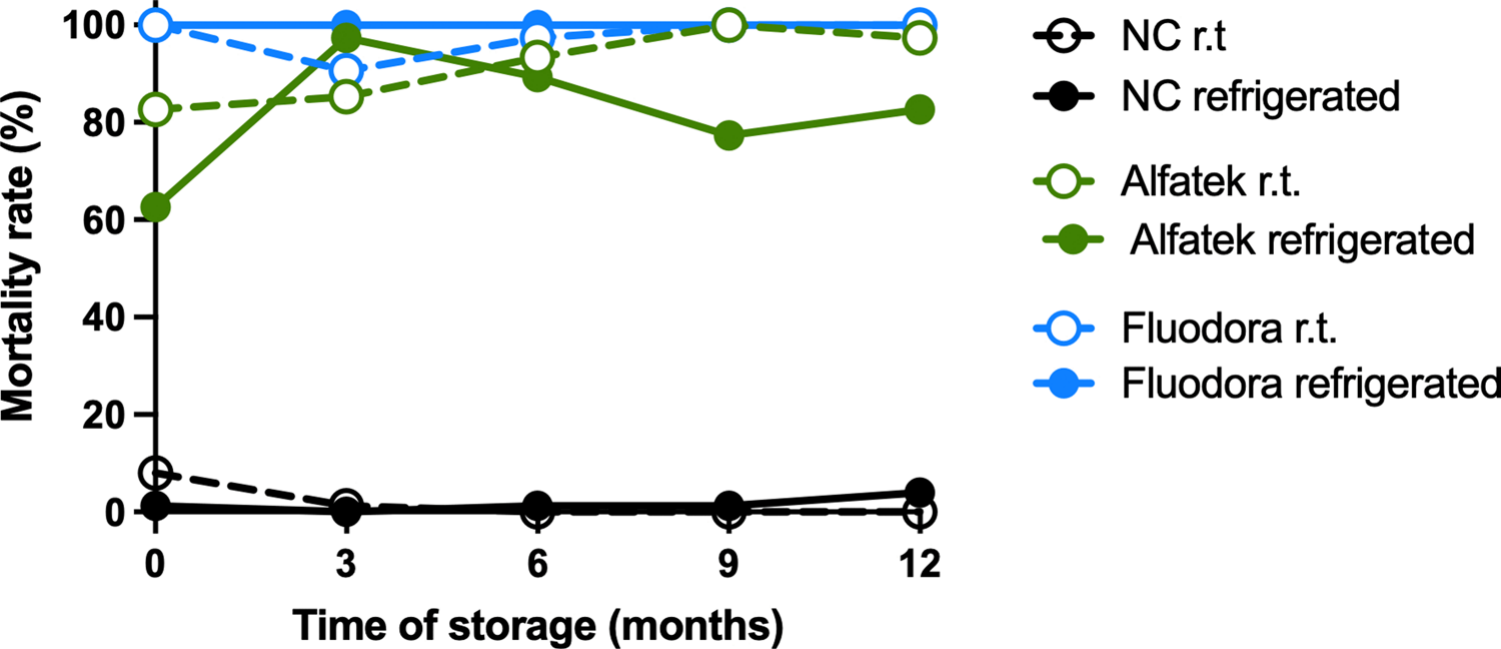
Effect of storage temperature on the stability of insecticide-impregnated papers over time in laboratory bioassays. Blue—Fludora FusionPM (test insecticide); Green—Alfatek 200SC (reference); Black—Negative control (water). Filled dots and continuous lines—papers stored under refrigeration; open dots and dashed lines—papers stored at room temperature. A total of *Lu. longipalpis* females were transferred per cone, in a total of 75 specimens per test sample (triplicate). Study period: September 2022 to October 2023

The impact of storage temperature on the residual efficacy of insecticide-impregnated papers over time, can be visualized in Fig. 5.

**Fig. 6:**
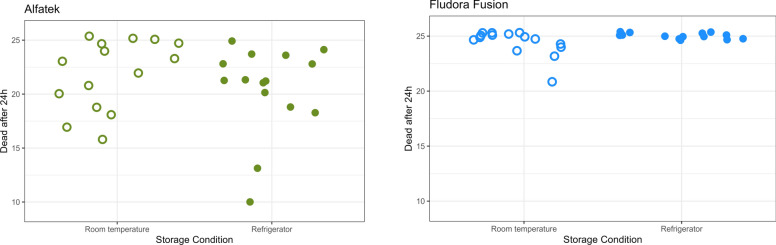
Effect of the storage temperature on the stability of papers impregnated with Fludora FusionPM in laboratory bioassays, along the period of study. Each dot represents one cone with 25 *Lu. longipalpis* females. Total: 15 cones per test sample (triplicates at 0, 3, 6, 9, and 12 months). X-axis represent the storage temperature (open dots- room temperature, filled dots- refrigerated). Blue dots- Fludora FusionPM; green dots- Alfatek200SC (reference)

Mortality rates of *Lu. longipalpis* exposed to insecticide-impregnated papers stored at room temperature or under refrigeration were statistically compared using the Mann–Whitney *U* test for independent samples. No difference was evidenced for Alfatech 200SC-impregnated papers under any storage condition (*U* = 89.5, *Z* = −0.94, *P* = 0.346). In contrast, mortality rates were lower for Fludora FusionPM-impregnated papers stored at room temperature compared with refrigerated ones (*U* = 30, *Z* = −2.369, *P* = 0.018). A general analysis of data dispersion for the two temperature conditions indicated greater efficiency and consistency for Fludora FusionPM results compared with Alfatech 200SC (Fig. 6).

The effect of storage temperature on the residual efficiency of insecticide-impregnated papers after 1 year of impregnation and 1-year average mortality rates of *Lu. longipalpis* is summarized in Table 1.

**Table 1:** Interference of storage temperature of impregnated papers on the mortality rates of *Lu. longipalpis* in laboratory bioassays

Paper storage	Test sample	Mortality rate (%)	
		After 1 year	1-year average
Refrigerated	Alfatek^®^ 200SC	82.7 (62/75)	81.8 (307/375)
	Fludora^®^ FusionPM	100.0 (75/75)	100.0 (375/375)
	Negative control	4.0 (3/75)	1.6 (6/375)
Room temperature	Alfatek^®^ 200SC	97.3 (73/75)	91.7 (344/375)
	Fludora^®^ FusionPM	100.0 (75/75)	97.6 (366/375)
	Negative control	0 (0/75)	1.9 (7/375)

Papers were stored under refrigeration or at room temperature, protected from light. The ratios between parenthesis represent the number of dead specimens divided by the total number of specimens in the assay(s). Period of study: September 2022 to October 2023

### Field bioassays

*Lutzomyia longipalpis* females were also susceptible to Fludora^®^ FusionPM in the field assay. Mortality rates at the 24-h readings of *Lu. longipalpis* exposed to Fludora FusionPM or Alfatek 200SC were compared with the negative control using the Kruskal–Wallis test. A statistically significant difference was detected among the three groups (*H* = 73.43, df = 2, *P* < 0.0001). Subsequent pairwise comparisons using Dunn’s post-test with Bonferroni correction for multiple comparisons showed that mortality in the negative control was significantly lower than in both insecticide groups (Fludora FusionPM versus negative control: *Z* = −8.176, *P* < 0.0001; Alfatek 200SC versus negative control: *Z* = 5.685, *P* < 0.0001). In contrast, the comparison between Fludora FusionPM and Alfatek 200SC (*Z* = −4.659, *P* < 0.0001) indicated lower mortality in the Alfatek 200SC group.

**Fig. 7:**
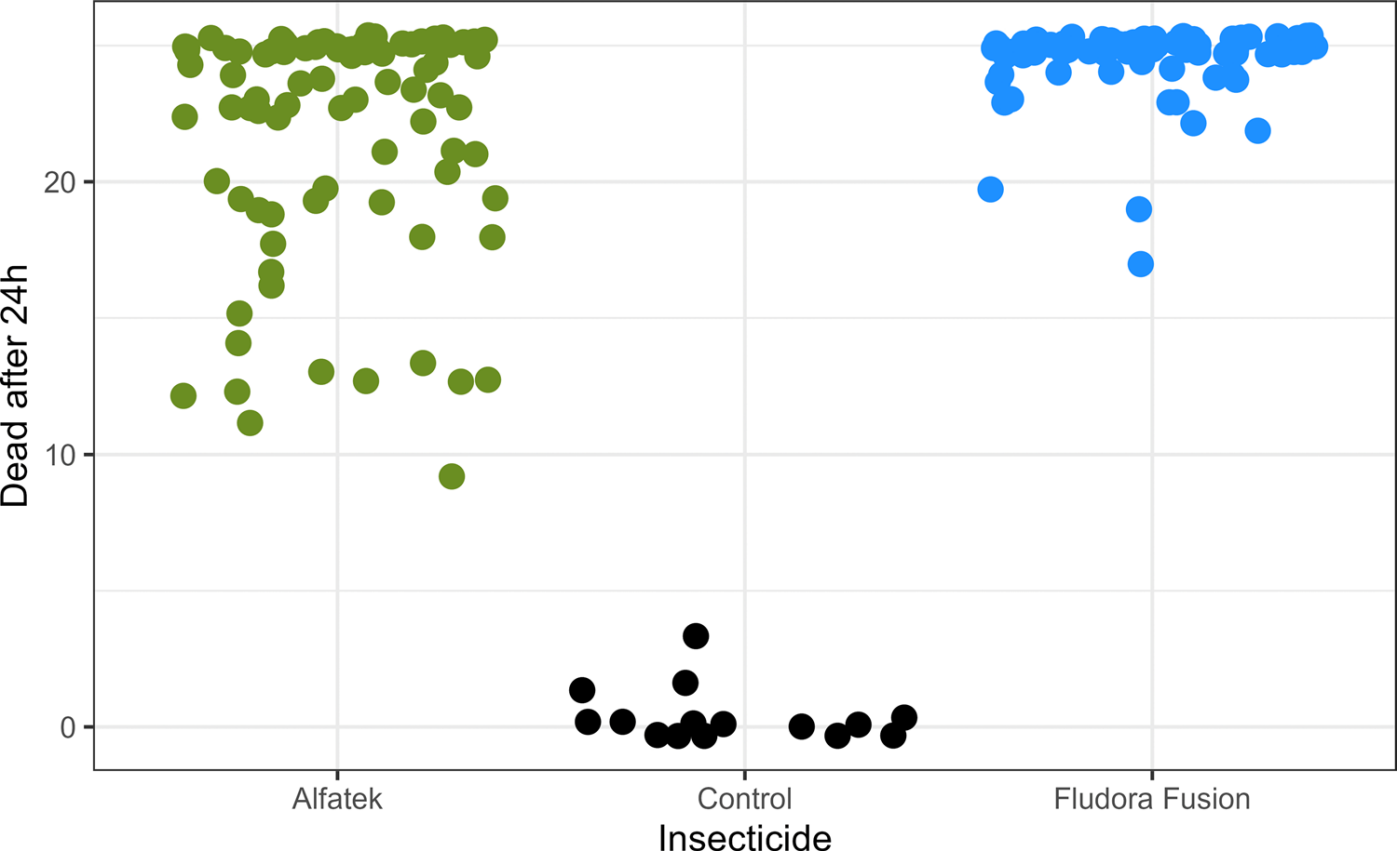
Susceptibility of *Lu. longipalpis* to Fludora FusionPM spraying in field bioassays, over 1 year. The cone assay was used. Each dot represents one experimental cone in a total of 45 cones for Fludora FusionPM and 44 cones for Alfatek 200SC. X-axis represent the insecticide or negative control (water). Black dots- negative control (water); blue dots- Fludora FusionPM; green dots- Alfatek 200SC. Reading time 24 h

The closer distribution of Fludora FusionPM data points indicate higher efficiency and consistency compared with Alfatek 200SC (Fig. 7). After 1 year of insecticide spraying, the mortality rates were 78.6% for Alfatek 200SC, 99.5% for Fludora FusionPM, and 0% for the negative control.

**Fig. 8:**
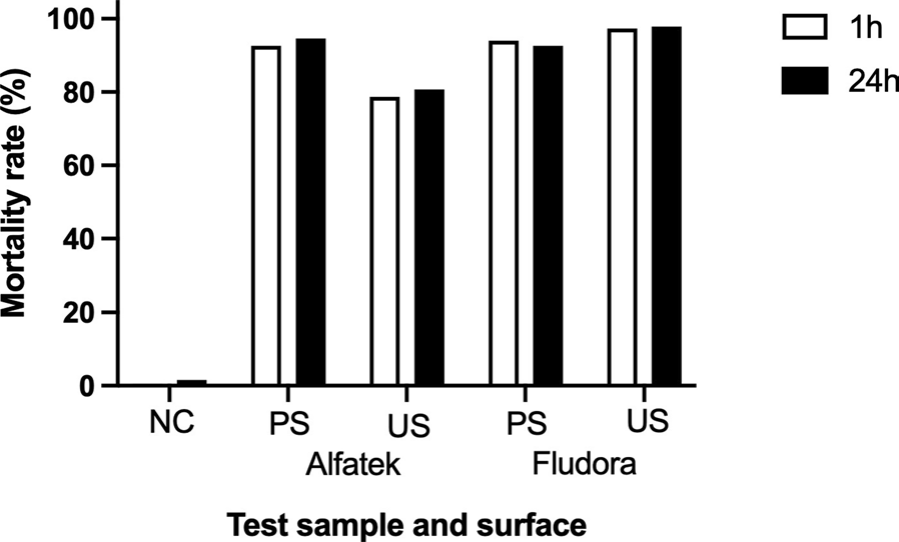
Interference of time reading on the susceptibility data of *Lu. longipalpis* to Fludora FusionPM in field bioassays. Average mortality rates were assessed 1 h (white bars) and 24 h (black bars) after exposition of the phlebotomine sand flies to insecticide-sprayed surfaces. *PS* painted masonry-plastered surface, *US* unpainted masonry-plastered surface, *NC* negative control. Period of study: February of 2023 to February of 2024

The impact of wall surface on the residual efficiency of the insecticides, was evaluated in PS and US masonry-plastered household walls. Similarly to laboratory assays, the mortality rates of *Lu. longipalpis* were evaluated after 1 h and 24 h of exposition (Fig. 8). The 24-h readings were adopted in further analysis to standardize laboratory and field procedures.

One cone in the Alfatek 200SC/US group at 9 months had partial data loss owing to sand fly adhering to tape. Therefore, analysis for this experimental condition was based on two cones (50 specimens) instead of three (75 specimens).

**Fig. 9:**
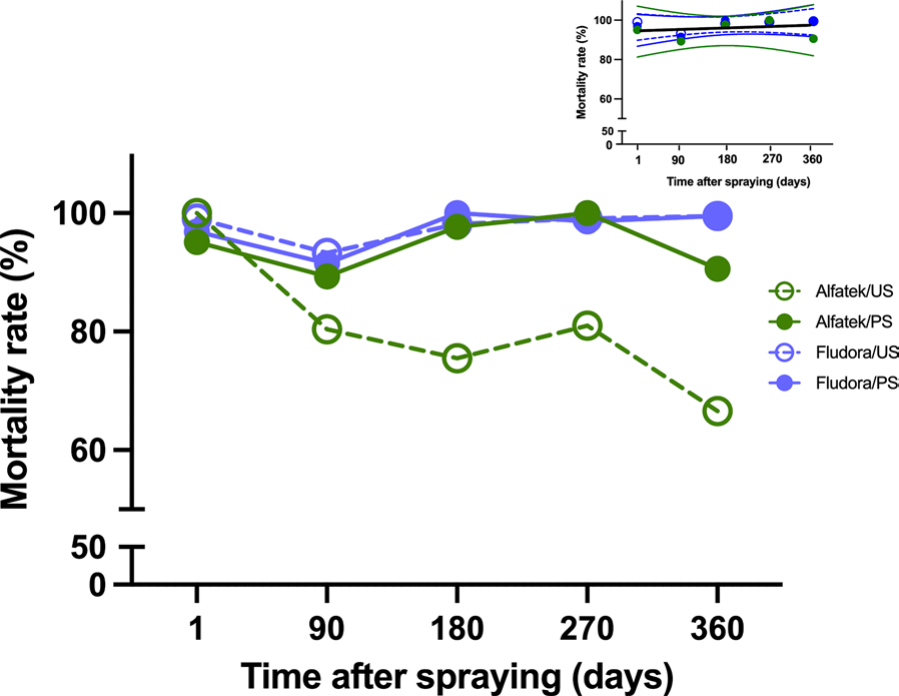
Residual effect of Fludora^®^ FusionPM spraying on painted (PS) and unpainted (UP) masonry-plastered household surfaces, in the field. The combinations insecticide/wall surface are indicated in the legend: Alfatek 200SC and painted masonry-plastered surface (PS); Alfatek 200SC and unpainted masonry-plastered surface (US); Fludora FusionPM and painted masonry-plastered surface (PS); Fludora^®^ FusionPM and unpainted masonry-plastered surface (US); Alfatek 200SC was the reference (positive control). Inset: 95% confidence intervals for the linear regression curves of Fludora^®^ FusionPM on PS and US surfaces, and of Alfatek^®^ 200SC on PS surfaces. The shared regression trend is depicted by the solid black line*.* Period of study: February of 2023 to February of 2024

Figure 9 shows the mortality rates over time according to the household surface type. The curve profiles for the four combinations (Fludora FusionPM on PS or US, and Alfatek 200SC on PS or US) were compared using simple linear regression. The null hypothesis was that all the lines had equal slopes. The analysis indicated that slope differences were significant (*F* = 5.431, DFn = 3, DFd = 12, *P* = 0.0136). Because the slopes differed substantially, it was not possible to validly test differences among intercepts.

Given the distinct pattern observed for Alfatek 200SC on US surfaces, these data were excluded, and the remaining three combinations were analyzed together. In this reduced dataset, the null hypothesis that a single regression line could be fitted to all observations was not rejected (*F* = 0.1846, DFn = 2, DFd = 9, *P* = 0.8345). Each of the three combinations was adequately fitted by a linear regression model with slopes not significantly different from zero (Table 2). Neither the slopes (*P* = 0.8345) nor the intercepts (*P* = 0.3367) differed significantly among the three curves. Because the slopes did not differ, a pooled value was calculated (0.007669). The intercepts were then compared and also found not to differ significantly (*F* = 1.204, DFn = 2, DFd = 11, *P* = 0.3367), allowing estimation of a pooled intercept (95.18). Based on these results, we concluded that the efficacy of Fludora FusionPM on PS and US surfaces, as well as that of Alfatek 200SC on PS, remained stable over time and could be represented by the shared equation *Y* = 0.007669 × *X* + 95.18. Table 2 summarizes the statistical parameters of the curve comparisons. In contrast, the efficacy of Alfatek 200SC on US declined progressively over time in a nonlinear manner, reaching a minimum of 66.7% after 1 year.

**Table 2:** Comparison of *Lu. longipalpis* mortality rates by insecticide on painted (PS) and unpainted (US) masonry-plastered household walls in field bioassays

Statistical parameters	Different curves for at least one data set			One curve for all data sets global (shared)
	Insecticide/household wall surface			
	Alfatek/PS	Fludora/US	Fludora/PS	
Best fit values				
Y-intercept	94.20	96.51	94.83	95.18
Slope	0.001904	0.007382	0.01372	0.007669
95% CI (profile likelihood)				
Y-intercept	81.22–107.2	89.78–103.2	86.70–103.0	91.63–98.73
Slope	−0.05697 to 0.06078	−0.02316 to 0.03792	−0.02313 to 0.05057	0.008422 to 0.02376
Goodness of fit				
Degrees of freedom	3	3	3	13
R squared	0.003519	0.1647	0.3189	0.07540
Sum of squares	82.80	22.28	32.43	174.5
Sy.x	5.254	2.725	3.288	3.664
Constraints				
Y-intercept				Y-intercept is shared
Slope				Slope is shared
Number of points				
*X* values	5	5	5	15
*Y* values analyzed	5	5	5	15
Is slope significantly non-zero?				
*F*	0.01059	0.5917	1.405	
DFn, DFd	1.3	1.3	1.3	
*P* value	0.9245	0.4978	0.3213	
Deviation from zero?	Not significant	Not significant	Not significant	

Linear regression equations represent the fitted mortality trends over time. Study period: February 2023 to February 2024

The 1-year average mortality rates of *Lu. longipalpis* exposed to insecticide-impregnated walls (Table 3) were compared using the Mann–Whitney *U* test for independent samples. No significant differences were observed for PS walls for either insecticide (*U* = 829.82, *Z* = −1.474, *P* = 0.141). In contrast, on US walls, mortality rates were significantly lower for Alfatek 200SC compared with Fludora FusionPM (*U* = 301.78, *Z* = 5.74, *P* < 0.0001).

**Table 3:** Interference of household wall surface on the mortality rates of *Lu. longipalpis* in field bioassays

Wall surface	Test sample	Mortality rate (%)	
		After 1 year	1-year average
PS	Alfatek^®^ 200SC	90.7 (204/225)	94.6 (1064/1125)
	Fludora^®^ FusionPM	99.5 (224/225)	97.3 (1095/1125)
US	Alfatek^®^ 200SC	66.7 (150/225)	80.7 (888/1100)
	Fludora^®^ FusionPM	99.1 (223/225)	97.9 (1101/1125)
Paper	Negative control	0 (0/75)	1.6 (6/375)

Painted (PS) or unpainted (US) masonry-plastered walls. The ratios between parenthesis represent the number of dead specimens divided by the total number of specimens in the assay(s). Period of study: February 2023 to February 2024

After 1 year of spraying, the mortality rates were 99.5% (224/225) and 90.7% (204/225) for Fludora FusionPM and Alfatek 200SC on PS, respectively. On US, Fludora FusionPM retained high residual activity (99.1%, 223/225) for the same time period (Table 3).

**Fig. 10:**
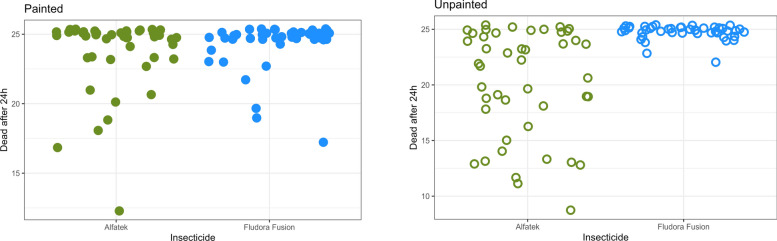
Mortality of *Lu. longipalpis* exposed to two different household surfaces, previously sprayed with insecticides, in the field. The insecticide spraying was tested on painted (PS) and unpainted (US) masonry-plastered surfaces. Each dot represents the number of dead insects per experimental cone previously filled with 25 females of *Lu. longipalpis* each. X-axis represent the insecticide. The insecticide spraying was tested on painted (filled dot) and unpainted (open dot) masonry-plastered surfaces. Blue dots- Fludora FusionPM; green dots- Alfatek 200SC (reference).Period of study: February of 2023 to February of 2024

The residual insecticide effect over 1 year is summarized in Fig. 10. The consistent efficiency of Fludora FusionPM on US walls under field conditions can be assumed from the dead insect clustering at the top quadrant on the right side of the corresponding figure.

With respect to adverse effects, one of the four residents in households treated with Fludora Fusion PM reported perceiving a faint odor, while another reported experiencing cutaneous pruritus. In households treated with Alfatek 200SC, one of the four residents perceived a faint odor, one reported cutaneous and ocular pruritus, another experienced ocular burning, and a fourth reported sneezing, coughing, and rhinorrhea. The ACE did not perceive any odor associated with either insecticide but reported a burning sensation on the face following the application of Alfatek 200SC. All adverse effects reported by household residents and the ACE are detailed in the Supplementary File.

## Discussion

The cost of insecticides and the logistics involved in spraying operations are two key challenges for large-scale application [16, 17]. Regarding phlebotomine sand flies, understanding mortality after exposure remains limited, highlighting the need to determine effective concentrations and residual durations to contain vector populations and mitigate resistance development [18, 19].

Insecticide selection for large-scale implementation depends not only on demonstrated vector mortality but also on safety for humans, animals, and the environment. Residual activity is critical to ensure sufficient persistency on treated surfaces, thereby disrupting vector life cycles and maintaining coverage until the next application [15, 20].

Laboratory bioassays demonstrated that Fludora FusionPM was highly toxic to *Lu. longipalpis* over 12 months, maintaining average efficacies > 90% throughout the trial. Hence, Fludora FusionPM could broaden the options for vector control programs targeting *Lu. longipalpis*.

In field assays, stratification by surface type revealed no significant difference for Fludora FusionPM or Alfatek 200SC on PS, whereas Fludora FusionPM exhibited significantly higher efficacy on US than Alfatek 200SC. This demonstrates the importance of insecticide–surface interactions in residual efficacy and emphasizes that formulation choice should consider wall type. Smooth, low-porosity surfaces favor suspension concentrate (SC) formulations such as Alfatek 200SC [21], which aligns with our observation that PS walls maintained high mortality for 12 months, whereas US walls retained efficacy for only 9 months.

Environmental conditions, including temperature, solar radiation, and rainfall, can also affect insecticide stability and persistence [22, 23]. Thus, context-specific spraying cycles may be necessary, particularly in endemic or epidemic areas.

Wettable powder (PM) formulations, such as Fludora FusionPM, are generally more effective on porous substrates, explaining their higher residual activity on both PS and US surfaces throughout the 12-month period. Previous studies with Fludora FusionPM on malaria vectors reported residual efficacy of 7–10 months depending on environmental conditions and surface types [24–26]. Ngufor et al. [27] found mortality rates above 90% for 12 months even in pyrethroid-resistant *Anopheles* populations when deltamethrin and clothianidin were combined, further supporting the benefits of mixed active ingredients.

This investigation required substantial logistical effort and comprehensive sampling, and we did not identify methodological limitations that could compromise the interpretation of the efficacy findings. However, the limited number of respondents available to report potential adverse effects represents a constraint, and additional studies with broader community participation are required.

Insecticide mixtures in integrated vector management can delay resistance development compared with single active ingredient formulations [11]. The combination of neonicotinoid and pyrethroid in Fludora FusionPM represents a promising strategy to reduce selective pressure on vector populations [26] and allow a single formulation to target multiple disease vectors, reducing environmental contamination and operational complexity.

Overall, the persistence of VL in endemic areas, urbanization of transmission, and reemergence of the disease underscore the need to reevaluate current control strategies [4, 28, 29]. Indoor residual spraying with effective insecticides can reduce *Lu. longipalpis* populations inside households. Given that the Brazilian Ministry of Health already uses Fludora FusionPM for mosquito control, its proven efficacy against sand flies could optimize vector control operations by allowing a single insecticide to target multiple vectors simultaneously. This approach supports insecticide rotation, resistance management, operational cost savings, and improved vector control effectiveness.

## Conclusions

This study is the first to assess the susceptibility of *Lu. longipalpis* to a pyrethroid–neonicotinoid combination (Fludora FusionPM), a formulation already employed in mosquito control. Laboratory bioassays showed that Fludora FusionPM was as effective as Alfatek 200SC in inducing sand fly mortality, under the test conditions. Field experiments revealed that surface type significantly affected insecticide performance: both products displayed similar efficacy on painted surfaces, whereas Fludora FusionPM achieved higher mortality and longer residual activity on unpainted walls under field test conditions. These findings highlight the importance of selecting insecticide formulations according to household surface characteristics to optimize operational effectiveness. Combinations of active ingredients with complementary modes of action may further strengthen VL surveillance and control programs by expanding rotation options, reducing the risk of resistance development, and improving overall vector management strategies.

## Supplementary Information

Additional file 1.

## Data Availability

All data supporting the conclusions of the article are included within this article and its additional file.

## References

[1] WHO (World Health Organization). Global vector control response 2017–2030. Geneva: World Health Organization; 2017. ISBN: 978-92-4-151297-8

[2] Young DG, Duncan MA. Guia para a identificação e distribuição geográfica de flebotomíneos *Lutzomyia* no México, Índias Ocidentais, América Central e do Sul (Diptera: Psychodidae). Mem Am Entomol Inst. 1994;54:881.

[3] Lopes JV, Michalsky EM, Pereira NCL, de Paula AJV, Lara-Silva FO, Silva-Lana R, et al. Entomological studies in Itaúna, Brazil, an area with visceral leishmaniasis transmission: fauna survey, natural *Leishmania* infection, and molecular characterization of the species circulating in phlebotomine sand flies (Diptera: Psychodidae). J Med Entomol. 2019;56:1368–76. 10.1093/jme/tjz061. 31121044 10.1093/jme/tjz061

[4] Rocha MF, Michalsky ÉM, Lara-Silva FO, Pereira NCL, Lana RS, França-Silva JC, et al. Impact of vector control actions in the abundance of *Lutzomyia longipalpis* in Montes Claros, Brazil. Acta Trop. 2022;228:106305. 10.1016/j.actatropica.2022.106305. 34998997 10.1016/j.actatropica.2022.106305

[5] Jeschke P, Nauen R, Schindler M, Elbert A. Overview of the status and global strategy for neonicotinoids. J Agric Food Chem. 2011;59:2897–908. 10.1021/jf101303g. 20565065 10.1021/jf101303g

[6] Simon-Delso N, Amaral-Rogers V, Belzunces LP, Bonmatin JM, Chagnon M, Downs C, et al. Systemic insecticides (neonicotinoids and fipronil): trends, uses, mode of action and metabolites. Environ Sci Pollut Res Int. 2015;22:5–34. 10.1007/s11356-014-3470-y. 25233913 10.1007/s11356-014-3470-y PMC4284386

[7] WHO (World Health Organization). Specifications for public health pesticides. WHO specifications and evaluations for public health pesticides: clothianidin + deltamethrin. 2021. https://extranet.who.int/prequal/sites/default/files/vcp-documents/WHOVC-SP_Clothianidin+Deltamethrin_2021.1.pdf. Accessed 20 Aug 2025.

[8] Ohkawara Y, Akayama A, Matsuda K, Andersch W. Clothianidin: a novel broad-spectrum neonicotinoid insecticide. In: The BCPC Conference—Pests & Diseases 2002: Proceedings of an International Conference, Volume 1: New Compounds and Uses for Pest Management. Farnham (UK): British Crop Protection Council; 2002. 51–58.

[9] Brasil. Technical Note No. 5/2020 – CGARB/DEIDT/SVS/MS). https://www.gov.br/saude/pt-br/centrais-de-conteudo/publicacoes/notas-tecnicas/2020/nota-tecnica-no-52020-cgarbdeidtsvsms.pdf/view. Accessed on 20 Aug 2025.

[10] WHO (World Health Organization). Global Malaria Programme. Global plan for insecticide resistance management in malaria vectors (GPIRM). Geneva: WHO; 2012.

[11] South A, Hastings IM. Insecticide resistance evolution with mixtures and sequences: a model-based explanation. Malar J. 2018;17:80. 10.1186/s12936-018-2203-y. 29448925 10.1186/s12936-018-2203-y PMC5815191

[12] Pessoa GC, Lopes JV, Rocha MF, Pinheiro LC, Rosa AC, Michalsky ÉM, et al. Baseline susceptibility to alpha-cypermethrin in *Lutzomyia longipalpis* (Lutz & Neiva, 1912) from Lapinha Cave (Brazil). Parasit Vectors. 2015;8:469. 10.1186/s13071-015-1076-y. 26381242 10.1186/s13071-015-1076-y PMC4573933

[13] Brasil. Guia de vigilância em saúde: volume 2. Ministério da Saúde, Departamento de Articulação Estratégica de Vigilância em Saúde e Ambiente. – 6. ed. – Brasília: Ministério da Saúde, 2023. 3v.:il. https://bvsms.saude.gov.br/bvs/publicacoes/guia_vigilancia_saude_v2_6ed.pdf. ISBN 978-65-5993-505-5. Accessed on 20 Aug 2025.

[14] R Core Team. R: A language and environment for statistical computing. R Foundation for Statistical Computing, Vienna. 2016. https://www.R-project.org. Accessed on 20 Aug 2025.

[15] WHO (World Health Organization). Standard operating procedure for testing the susceptibility of adult sand flies to insecticides in WHO tube tests. 2023. SOP version no. WHO sand fly tube tests/NTD/2023/01. ISBN 978-92-4-007462-0 (electronic version). https://iris.who.int/bitstream/handle/10665/370364/9789240074620-eng.pdf?sequence=1. Accessed 20 Aug 2025.

[16] Chabi J, Seyoum A, Edi CVA, Kouassi BL, Yihdego Y, Oxborough R, et al. Efficacy of partial spraying of SumiShield, Fludora Fusion and Actellic against wild populations of *Anopheles gambiae* s.l. in experimental huts in Tiassalé, Côte d’Ivoire. Sci Rep. 2023;13:11364. 10.1038/s41598-023-38583-y. 37443329 10.1038/s41598-023-38583-y PMC10344869

[17] Obembe A, Oduola AO, Adeogun A, Inyang U, Oyeniyi T, Olakiigbe A, et al. Implementation of malaria vector surveillance and insecticide resistance monitoring interventions in Nigeria. Glob Health Res Policy. 2024;9:55. 10.1186/s41256-024-00397-4. 39741286 10.1186/s41256-024-00397-4 PMC11686922

[18] Alexander B, Barros VC, de Souza SF, Barros SS, Teodoro LP, Soares ZR, et al. Susceptibility to chemical insecticides of two Brazilian populations of the visceral leishmaniasis vector *Lutzomyia longipalpis* (Diptera: Psychodidae). Trop Med Int Health. 2009;14:1272–7. 10.1111/j.1365-3156.2009.02371.x. 19772549 10.1111/j.1365-3156.2009.02371.x

[19] Chowdhury R, Dotson E, Blackstock AJ, McClintock S, Maheswary NP, Faria S, et al. Comparison of insecticide-treated nets and indoor residual spraying to control the vector of visceral leishmaniasis in Mymensingh District, Bangladesh. Am J Trop Med Hyg. 2011;84:662–7. 10.4269/ajtmh.2011.10-0682. 21540372 10.4269/ajtmh.2011.10-0682 PMC3083730

[20] Corrêa APSA, Galardo AKR, Lima LA, Câmara DCP, Müller JN, Barroso JFS, et al. Efficacy of insecticides used in indoor residual spraying for malaria control: an experimental trial on various surfaces in a “test house.” Malar J. 2019;18:345. 10.1186/s12936-019-2969-6. 31601226 10.1186/s12936-019-2969-6 PMC6785876

[21] WHO (World Health Organization). Operational manual on leishmaniasis vector control, surveillance, monitoring and evaluation. 2022. ISBN 978-92-4-006034-0 (electronic version). https://www.who.int/publications/i/item/9789240060340. Accessed 20 Aug 2025.

[22] Chaumeau V, Wisisakun P, Sawasdichai S, Kankew P, Htoo GN, Saithanmettajit S, et al. Longevity of the insecticidal effect of three pyrethroid formulations applied to outdoor vegetation on a laboratory-adapted colony of the Southeast Asian malaria vector *Anopheles dirus*. PLoS ONE. 2020;15:e0231251. 10.1371/journal.pone.0231251. 32287300 10.1371/journal.pone.0231251 PMC7156039

[23] Rhodes LA, McCarl BA. An analysis of climate impacts on herbicide, insecticide, and fungicide expenditures. Agronomy. 2020;10:745. 10.3390/agronomy10050745.

[24] Agossa FR, Padonou GG, Fassinou AJYH, Odjo EM, Akuoko OK, Salako A, et al. Small-scale field evaluation of the efficacy and residual effect of Fludora® Fusion (mixture of clothianidin and deltamethrin) against susceptible and resistant *Anopheles gambiae* populations from Benin, West Africa. Malar J. 2018;17:484. 10.1186/s12936-018-2633-6. 30594207 10.1186/s12936-018-2633-6 PMC6311023

[25] Fuseini G, Phiri WP, von Fricken ME, Smith J, Garcia GA. Evaluation of the residual effectiveness of Fludora™ fusion WP-SB, a combination of clothianidin and deltamethrin, for the control of pyrethroid-resistant malaria vectors on Bioko Island, Equatorial Guinea. Acta Trop. 2019;196:42–7. 10.1016/j.actatropica.2019.05.006. 31077641 10.1016/j.actatropica.2019.05.006

[26] Fongnikin A, Houeto N, Agbevo A, Odjo A, Syme T, N’Guessan R, et al. Efficacy of Fludora® Fusion (a mixture of deltamethrin and clothianidin) for indoor residual spraying against pyrethroid-resistant malaria vectors: laboratory and experimental hut evaluation. Parasit Vectors. 2020;13:466. 10.1186/s13071-020-04341-6. 32917255 10.1186/s13071-020-04341-6 PMC7488472

[27] Ngufor C, Fongnikin A, Rowland M, N’Guessan R. Indoor residual spraying with a mixture of clothianidin (a neonicotinoid insecticide) and deltamethrin provides improved control and long residual activity against pyrethroid resistant *Anopheles gambiae* sl in Southern Benin. PLoS ONE. 2017;12:e0189575. 10.1371/journal.pone.0189575. 29252986 10.1371/journal.pone.0189575 PMC5734732

[28] Davies CR, Llanos-Cuentas A, Canales J, Leon E, Alvarez E, Monge J, et al. The fall and rise of Andean cutaneous leishmaniasis: transient impact of the DDT campaign in Peru. Trans R Soc Trop Med Hyg. 1994;88:389–93. 10.1016/0035-9203(94)90395-6. 7570813 10.1016/0035-9203(94)90395-6

[29] Barata RA, Michalsky EM, Fujiwara RT, França-Silva JC, Rocha MF, Dias ES. Assessment of sand fly (Diptera, Psychodidae) control using cypermethrin in an endemic area for visceral leishmaniasis, Montes Claros, Minas Gerais State. Cad Saude Publica. 2011;27:2117–23. 10.1590/s0102-311x2011001100005. 22124489 10.1590/s0102-311x2011001100005

