# Supplementary material for: Susceptibility of Lutzomyia longipalpis (Lutz & Neiva, 1912) to Fludora FusionPM, a combination of clothianidin and deltamethrin: field and laboratory bioassays
Source: Parasit Vectors. 2025 Dec 24;19:49. doi: 10.1186/s13071-025-07206-y (PMC12849672; doi:10.1186/s13071-025-07206-y)
Supplement: Supplementary file 1 — Additional file 1. [file 13071_2025_7206_MOESM1_ESM.docx]

**Supplementary file**

**Questionnaire applied and answers by residents 24 h after the insecticide spraying**

1. How long do you live here? Your domicile is frequently sprayed with insecticides? At what frequency and for what reason?

| HU number | Time of residence (years) | Periodical insecticide spraying in the  residence | |
| --- | --- | --- | --- |
|  |  | Yes | No |
| 1 | 7 |  | X |
| 2 | 16 |  | X |
| 3 | 10 |  | X |
| 4 | 17 |  | X |
| 5 | 6 |  | X |
| 6 | 76 |  | X |
| 7 | 26 |  | X |
| 8 | 7 |  | X |
| 9 | 14 |  | X |

1. What is your perception/opinion about insecticides spraying and use of insecticides in domiciles? How would you describe the characteristics of the insecticide sprayed in your house? (Pause). Did you notice any smell? If so, persistent or transitory? Strong or faint? Is the smell annoying?

Among four residents from HU 1, 2, 3 and 4 (sprayed with Fludora® Fusion PM), one related a faint smell. Similarly, one among four residents from HU’s sprayed with Alfatek® 200SC noticed some smell. Another resident related eye’s irritation.

1. Do you have any health problem?

Seven residents related no health problem, one resident reported high blood pressure and knee problem, and one resident did not answer the question.

1. Did you feel any previous (before the study) physical discomfort associated to the use of insecticides? (Pause) Allergy, irritations? To what insecticide?

| HU number | Insecticide sprayed | Previous reactions to other insecticides | | |
| --- | --- | --- | --- | --- |
|  |  | Yes | No | No answer or do not know |
| 1 | Fludora® FusionPM | X |  |  |
| 2 |  | X |  |  |
| 3 |  | X |  |  |
| 4 |  |  |  | X |
| 5 | Alfatek® 200SC |  |  | X |
| 6 |  |  | X |  |
| 7 |  |  |  | X |
| 8 |  |  |  | X |
| 9 |  |  | X |  |

1. In the present study, after the insecticide spraying, did you feel any airway irritation (sneezing, coughing?). Did you notice any of these reactions in the ACE?

None of the residents participating in the study noticed any airway irritation. One resident reported that her mother (non-participant) started to sneeze. No reaction observed in the ACE.

| HU number | Insecticide sprayed | Location and type of symptom | | |
| --- | --- | --- | --- | --- |
|  |  | Eye | Skin | Airway |
| 1 | Fludora® FusionPM |  | X (itching) |  |
| 2 |  |  |  |  |
| 3 |  |  |  |  |
| 4 |  |  |  |  |
| 5 | Alfatek® 200SC | X (itching) | X (itching) |  |
| 6 |  |  |  |  |
| 7 |  |  |  |  |
| 8 |  |  |  | X (sneezing, coughing, runny nose) |
| 9 |  | X (burning) |  |  |

1. The insecticide spraying causes any effect on the treated surfaces, like stains? If so, is it easily cleanable?

None of the residents reported any effect on the sprayed walls.

1. Do you have any additional comment or observation about the insecticide or any other aspect that you judge relevant?

No.

**Questionnaire applied to the CEA at the end of insecticide spraying (February/2023)**

1. How long have you been working as ACE? Always in this municipality or not?

The ACE has been working for 28 years in the same municipality.

1. Did you receive any previous training on the use of sprinklers and spray pumping in domiciles? DO you have any previous experience on that?

Yes, the ACE was trained for the task.

1. Did you have any health problem, at any time, related to your professional activity?

No.

1. How would you describe the characteristics of the insecticides sprayed by you? (Pause) Did you notice any smell? If so, persistent or transitory? Strong or faint? Is the smell annoying?

The ACE did not report any smell.

1. Did you feel any previous discomfort associated to the use of insecticides? (Pause) Allergy, skin irritation? To what insecticide?

No.

1. Did you feel any skin irritation such as redness or itch?

The ACE reported strong burning sensation in the skin only with Alfatek.

1. The insecticide spraying causes any effect on the treated surfaces, like stains? If so, is it easily cleanable?

No effect on the walls.

1. Do you have any additional comment or observation about the insecticide or any other aspect that you judge relevant?

No.

| Question | Alfatek^®^200SC | | Fludora^®^FusionPM | |
| --- | --- | --- | --- | --- |
|  | Yes | No | Yes | No |
| Any smell during/after the spraying |  | X |  | X |
| Physical discomfort during/after the spraying | X^a^ |  |  | X |
| Respiratory tract irritation during/after the spraying |  | X |  | X |
| Appearance of stains in the wall surface after spraying |  | X |  | X |

^a^ Burning sensation in the face
